# Awareness and acceptance of latent tuberculosis infection screening and preventive treatment among healthcare workers: a cross-sectional study in Chongqing, China

**DOI:** 10.3389/fpubh.2026.1770064

**Published:** 2026-06-24

**Authors:** Yalan Liu, Ke Huang, Fangyu Lin, Jianfeng Zhu, Yuxin Zhang, Peiming Cao, Wenwen Deng, Bing Deng

**Affiliations:** 1 Infectious Disease Prevention and Control Office, Chongqing Public Health Medical Center, Chongqing, China; 2 Department of Medical, Chongqing Public Health Medical Center, Chongqing, China

**Keywords:** acceptance, healthcare workers (HCWs), knowledge, latent tuberculosis infection (LTBI), tuberculosis preventive treatment (TPT)

## Abstract

**Objective:**

Healthcare workers (HCWs) are at high risk for latent tuberculosis infection (LTBI), yet screening and tuberculosis prevention treatment (TPT) rates remain low. This study aims to assess acceptance of LTBI screening and TPT among HCWs and identify factors influencing their willingness.

**Methods:**

A stratified random sampling was conducted across 31 districts, involving 46 tuberculosis (TB) designated institutions in Chongqing. HCWs were surveyed using a structured questionnaire to assess their knowledge, attitudes, and acceptance regarding LTBI screening and TPT.

**Results:**

Among 1,022 HCWs, 39.7% reported limited LTBI knowledge, and 67.4% had never undergone LTBI screening. Willingness rates for screening and TPT were 78.7 and 93.3%, respectively. Among those unwilling to undergo screening, 87.6% cited reasons such as perceiving it as unnecessary or lacking time, while 57.8% expressed concerns about the inspection process and associated cost. Additionally, 79.4% of those unwilling to accept TPT cited fear of adverse drug reactions. Department was significantly associated with willingness to undergo screening (*p* = 0.003), while profession (*p* = 0.001) and work experience (*p* = 0.016) were significantly associated with willingness to receive TPT. Multivariable analysis revealed that prior LTBI screening history (OR = 5.145), history of treating TB patients (OR = 1.518), and support for TPT (OR = 1.497) were independent predictors of screening acceptance, while support for TPT was the sole independent predictor of TPT acceptance (OR = 7.110).

**Conclusion:**

Strengthening health education, providing policy and financial support, and targeting high-risk departments for screening and TPT may improve compliance among HCWs. Additionally, fostering positive attitudes toward TPT and ensuring positive initial screening experiences are critical to enhancing acceptance.

## Introduction

Tuberculosis (TB) remains a leading cause of mortality globally, and China is one of the high-burden countries, ranking third in the number of TB cases (1). Latent tuberculosis infection (LTBI) represents a significant reservoir for potential TB cases (2), without intervention, 5–10% of individuals with LTBI are at risk of progressing to active TB during their lifetime (3, 4). China has a substantial burden of LTBI, reporting a prevalence rate of 13 to 40% (5–7). Given the high prevalence, preventive measures such as tuberculosis preventive treatment (TPT) are essential to reduce the risk of progression to active TB (8, 9).

Different countries and regions have implemented LTBI screening programs targeting high-risk groups, including close contacts of TB patients, people living with HIV, students, and healthcare workers (HCWs) (10–15). The prevalence of LTBI varies significantly across regions and populations. A meta-analysis of 31,431 HCWs from 25 low-incidence countries revealed that LTBI rates ranged from 0.9 to 85.5%, indicating a year-by-year increase in LTBI rates among HCWs (16). In both high-burden and low-burden countries, HCWs face a higher risk of LTBI compared to the general population (17).

While at work, HCWs are frequently exposed to TB patients who may spread the infection in hospitals even before their diagnosis is confirmed (18). Therefore, systematic screening and treatment of LTBI among HCWs are critical for TB prevention and control. In China, a meta-analysis involving 9,654 HCWs reported an LTBI rate of 51.5%. Another meta-analysis involving 2,991 HCWs engaged in TB prevention and control found infection rates of 29.5% for T-SPOT, 41.1% for QFT, and 43.4% for TST (19). Studies have shown that TPT initiation rates range from 15 to 98.7%, with most rates below 60%, while completion rates range from 16 to 97.3% among those who start TPT, with most completion rates also below 60% (20, 21).

In China, both the *National TB Prevention and Control Plan (2024–2030)* and the *technical specifications for TB prevention and control* issued by the National Health Commission recommend LTBI screening and TPT for high-risk populations but do not mandate these interventions for HCWs. Consequently, LTBI screening and TPT implementation rates among HCWs remain low and are not widely adopted. Currently, limited data exist on HCW compliance with LTBI screening and TPT, and their awareness of LTBI as well as acceptance of screening and TPT remain unclear. Clarifying the acceptance of LTBI screening and TPT among HCWs and identifying influencing factors are critical for implementing effective LTBI interventions. Given the pivotal role of LTBI awareness in shaping acceptance, this study aims to assess HCWs’ knowledge of LTBI, their willingness to undergo screening and TPT, and explore associated influencing factors in designated TB medical institutions in Chongqing, China.

## Methods

### Study design

A cross-sectional study design was employed to assess the awareness and acceptance of LTBI screening and TPT among HCWs in Chongqing, China. This study was conducted by the Chongqing Tuberculosis Medical Quality Control Center from August to October 2024.

### Population and sampling

**Figure 1:**
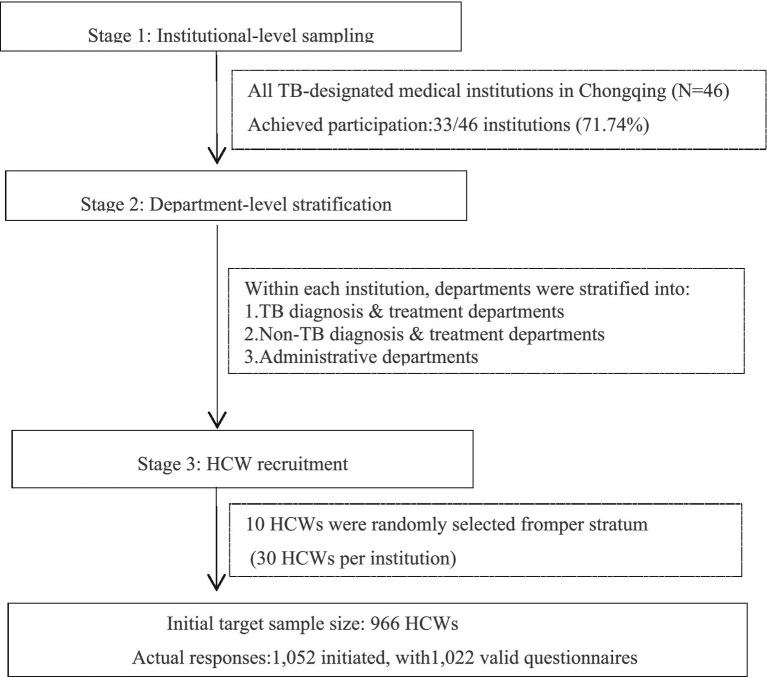
Schematic diagram of the sampling strategy.

The target population for this study comprised HCWs employed in TB-designated medical institutions within Chongqing, including physicians, nurses, technicians, managers and support staff. A stratified random sampling technique was employed to ensure adequate representation of participants across different departments, thereby enhancing the generalizability of the findings among various categories of HCWs (Figure 1).

In the first stage, all 46 TB-designated medical institutions in Chongqing were enrolled. In the second stage, departments within each participating institution were stratified into three strata: TB diagnosis and treatment departments, non-TB diagnosis and treatment departments, and administrative departments. In the third stage, ten HCWs were randomly recruited from each stratum (30 HCWs per institution). The initial target sample was calculated as 966, based on an anticipated 70% response rate (46 institutions × 30 HCWs × 70%). A total of 1,022 valid questionnaires were collected from 1,052 initiated responses, representing a 97.15% completion rate. The final sample achieved a 71.74% institutional participation rate (33 out of 46 institutions).

### Study process

A structured questionnaire was carefully developed to measure HCWs’ awareness and acceptance concerning LTBI screening and TPT. First, a preliminary questionnaire was drafted based on existing literature and relevant guidelines. To ensure its relevance and content validity, five experts in nosocomial infection prevention and epidemiology were invited to review and refine the questionnaire item by item. Following expert review, semi-structured interviews were conducted with TB specialists to gain insights into the current challenges and practices related to LTBI screening and TPT implementation, as well as potential barriers faced by HCWs. These interviews provided valuable input, which informed further revisions and optimization of the questionnaire. Subsequently, a pilot survey was conducted with seven HCWs selected through purposive sampling to represent diverse clinical roles (physicians, nurses, laboratory technicians), departments (TB-related and non-TB), and levels of experience (2–15 years). Participants were recruited from three hospital tiers and no prior involvement in questionnaire development. The average completion time was approximately five minutes, indicating high feasibility and acceptability. For the formal survey, the finalized questionnaire was distributed to the designated medical institutions via an online platform.

### Questionnaire contents

The self-designed electronic questionnaire, consisting primarily of closed-ended questions to facilitate quantitative analysis, collected data on the following domains:

- *Sociodemographic characteristics*: This section comprised of six items that gathered background data, including sex, age, education level, professional title, years of working experience and department.
- *Clinical exposure history:* This domain included four single-choice questions that assessed the HCWs’ exposure, specifically history of treating TB patients, close contact with TB patients, personal and family history of TB.

#### Knowledge level of LTBI and TPT

This domain through five items covering LTBI transmission, progression, and screening populations, as well as the target populations and treatment regimens for TPT. Each correct response was assigned a score of ‘1’, and incorrect were scored as ‘0’. The total possible score was 5. Based on their scores, participants’ knowledge levels were categorized as follows: no knowledge at all (scores≤1), limited knowledge (scores 2–3), familiarity (scores ≥4).

#### Attitudes toward LTBI screening and TPT

Eight items explored HCWs’ perceptions and acceptance, including preferences for LTBI screening and TPT methods, as well as reasons for not accepting screening or preventive treatment.

#### Practices in LTBI and TPT management

This section assessed practical behaviors through five questions that focused on HCWs’ practices and institutional care policies regarding TB/LTBI health examinations.

### Data collection and quality control

Participants completed the electronic questionnaire via a secure online platform. To ensure data integrity, several quality control measures were implemented. The electronic questionnaire was designed with mandatory completion of critical variables and embedded real-time validation checks. Two uniformly trained managers were responsible for standardizing data collection and conducted random manual audits of 10% responses for logical consistency.

### Ethical considerations

This study was conducted in accordance with the Declaration of Helsinki. Ethical approval was obtained from the Institutional Review Board of Chongqing Public Health Medical Center. Prior to data collection, all participants were provided with detailed information about the study’s objectives, confidentiality protocols, and their right to withdraw at any time without consequence. Informed consent was obtained from all participants prior to enrollment; only those who provided consent were granted access to the questionnaire.

### Statistical analysis

Descriptive statistics were presented as frequencies, proportions, and means. Chi-square test was employed to compare the differences in TB prevention and control behaviors, LTBI knowledge, and acceptance of LTBI screening and TPT across departments. Univariate analysis using chi-square tests was also applied to examine factors associated with LTBI screening and TPT acceptance. Binary logistic regression analysis was performed to identify significant predictors of LTBI screening and TPT acceptance, with adjustment for relevant sociodemographic and clinical factors. The statistically significant level was set at *p* < 0.05. All statistical analyses were performed using SPSS 26 (IBM Corp., Armonk, NY, United States).

## Results

### Demographic characteristics of the participants

In this study, 1,022 HCWs were surveyed, of whom 522 (51.8%) were TB-related HCWs engaged in the diagnosis and treatment of TB. The median age of the respondents was 35 years, with a female-to-male ratio of approximately 3.2:1. A total of 91.6% of the respondents held a bachelor’s degree or higher, and approximately 60.0% had an intermediate professional title or above. Approximately half of the respondents had more than 10 years of work experience. Detailed demographic characteristics are presented in Table 1.

**Table 1:** Demographic characteristics of the participants.

Characteristics	Variables	Frequency	Percentage
Sex	Male	246	24.1
	Female	776	75.9
Age (years)	≤25	61	6.0
	26–35	487	47.7
	36–45	306	29.9
	46–55	148	14.5
	≥56	20	2.0
Department	Infectious disease	317	31.0
	Respiratory department	98	9.6
	Laboratory department	54	5.3
	Radiology department	53	5.2
	Administrative department	182	17.8
	Other	318	31.1
Education level	Master degree or above	86	8.4
	Bachelor’s degree	777	76.0
	Associate degree and below	159	15.6
Professional title	Senior	185	18.1
	Intermediate	425	41.6
	Junior	348	34.1
	Other	64	6.3
Work experience (years)	≤5	244	23.9
	6–10	247	24.2
	≥11	531	52.0

### Awareness and acceptance of TB prevention, LTBI and TPT

As shown in Table 2, among TB-related HCWs, 76.1% reported having treated patients with TB, and 50.6% reported a history of close contact with TB patients without wearing a mask. Additionally, 5.7% of HCWs had been diagnosed with TB themselves, and 7.0% reported that a family member or relative had been diagnosed with TB. A total of 93.6% indicated that their units.

**Table 2:** Awareness and acceptance of TB prevention, LTBI, and TPT among HCWs from different departments.

Items	TB diagnosis and treatment departments	Non-TB related departments	Administrative departments	Total	χ^2^	*p*
Have you treated TB patients in the past two years?						
Yes	397 (76.1)	143 (45.0)	16 (8.8)	556 (54.4)	350.454	0.000
No	88 (16.9)	128 (40.3)	62 (34.1)	278 (27.2)		
Uninvolved	37 (7.1)	47 (14.8)	104 (57.1)	188 (18.4)		
Have you had close contact with TB patients without wearing a mask?						
Yes	264 (50.6)	107 (33.6)	48 (26.4)	419 (41.0)	42.985	0.000
No	258 (49.4)	211 (66.4)	134 (73.6)	603 (59.0)		
Have your family members or relatives been diagnosed with TB within 5 years?						
Yes	29 (5.6)	27 (8.5)	16 (8.8)	72 (7.0)	3.631	0.163
No	493 (94.4)	291 (91.5)	166 (91.2)	950 (93.0)		
Have you ever been diagnosed with TB?						
Yes	39 (7.5)	10 (3.1)	9 (4.9)	58 (5.7)	7.131	0.028
No	483 (92.5)	308 (96.9)	173 (95.1)	964 (94.3)		
Whether the units conducted annual health examination?						
Yes	490 (93.9)	295 (92.8)	172 (94.5)	957 (93.6)	0.682	0.711
No	32 (6.1)	291 (7.2)	10 (5.5)	65 (6.4)		
Did you have a history of TB-related examination?						
Yes	416 (79.7)	217 (68.2)	128 (70.3)	761 (74.5)	15.623	0.000
No	106 (20.3)	101 (31.8)	54 (29.7)	261 (25.5)		
What method do you choose in TB-related examination?						
Chest x-ray /CT	383 (92.1)	196 (90.3)	117 (91.4)	696 (91.5)	3.396	0.907
Tuberculin skin test	23 (5.5)	11 (5.1)	8 (6.3)	42 (5.5)		
Immune examination	5 (1.2)	5 (2.3)	2 (1.6)	12 (1.6)		
Sputum smear examination	3 (0.7)	3 (1.4)	1 (0.8)	7 (0.9)		
Other	2 (0.5)	2 (0.9)	0 (0.0)	4 (0.5)		
Do you know the definition, diagnosis, treatment and prevention of LTBI?						
Have no idea at all	18 (3.4)	10 (3.1)	10 (5.5)	38 (3.7)	159.973	0.000
Known a little bit	106 (20.3)	157 (49.4)	105 (57.7)	368 (36.0)		
Familiar with	398 (76.3)	151 (47.5)	67 (36.8)	616 (60.3)		
Have you been screened for LTBI?						
Yes	223 (42.7)	66 (20.8)	44 (24.2)	333 (32.6)	50.53	0.000
No	299 (57.3)	252 (79.2)	138 (75.8)	689 (67.4)		
Are you willing to undergo LTBI screening?						
Yes	434 (83.1)	239 (75.2)	131 (72.0)	804 (78.7)	13.146	0.001
No	88 (16.9)	79 (24.8)	51 (28.0)	218 (21.3)		
Which method do you choose to screen for LTBI?						
TST	290 (55.6)	220 (69.2)	122 (67.0)	632 (61.8)	27.220	0.000
EC	87 (16.7)	28 (8.8)	24 (13.2)	139 (13.6)		
IGRA	80 (15.3)	28 (8.8)	13 (7.1)	121 (11.8)		
Other	65 (12.5)	42 (13.2)	23 (12.6)	130 (12.7)		
Are you willing to receive TPT for LTBI?						
Yes	479 (91.8)	304 (95.6)	171 (94.0)	954 (93.3)	4.812	0.090
No	43 (8.2)	14 (4.4)	11 (6.0)	68 (6.7)		
Which preventive treatment method would you prefer?						
Chemotherapy	215 (44.9)	131 (43.1)	79 (46.2)	425 (44.5)	0.472	0.790
Immunological therapy	264 (55.1)	173 (56.9)	92 (53.8)	529 (55.5)		
Does the units have a care policy for HCWs with TB?						
No	336 (64.4)	166 (52.2)	103 (56.6)	605 (59.2)	12.732	0.002
Yes	186 (35.6)	152 (47.8)	79 (43.4)	417 (40.8)		
Do you support screening for LTBI in high-risk position?						
Yes	505 (96.7)	308 (96.9)	181 (99.5)	994 (97.3)	3.996	0.136
No	17 (3.3)	10 (3.1)	1 (0.5)	28 (2.7)		
Do you support TPT for LTBI?						
Yes	412 (78.9)	260 (81.8)	159 (87.4)	831 (81.3)	6.381	0.041
No	110 (21.1)	58 (18.2)	23 (12.6)	191 (18.7)		

conducted annual healthy examination. Of these, 74.5% reported undergoing TB-related examinations, with 91.5% choosing X-ray/CT and only 8.0% opting for LTBI screening.

Furthermore, 39.7% of HCWs reported limited LTBI knowledge, and 67.4% reported had never undergone LTBI screening. The willingness to undergo LTBI screening and TPT were 78.7 and 93.3%, respectively.

### Barriers to LTBI screening and TPT acceptance

**Figure 2:**
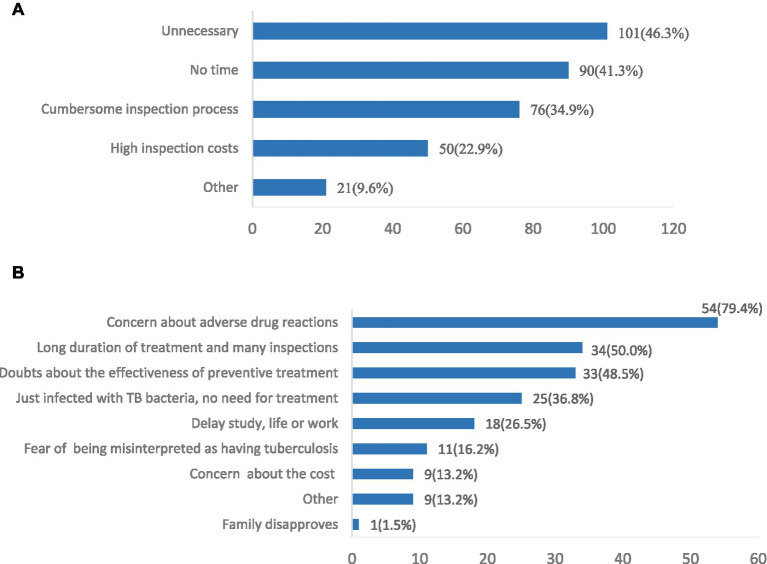
Concerns and barriers associated with LTBI screening and TPT among HCWs. **(A)** Reasons for refusing LTBI screening (*n* = 218). **(B)** Reasons for refusing TPT (*n* = 68).

As shown in Figure 2A, among the 218 HCWs unwilling to undergo LTBI screening, the most frequently cited barriers included perceiving it as unnecessary (46.3%, 101/218), lack of time (41.3%, 90/218), and concerns related to the inspection process and associated costs (57.8%, 126/218). Among the 68 HCWs unwilling to undergo TPT, the predominant concern was adverse drug reactions (79.4%, 54/68), followed by the perceived long duration of treatment (50.0%, 34/68), doubts about the effectiveness of TPT (48.5%, 33/68), and concerns regarding the cost of treatment and inspections (13.2%, 9/68) (Figure 2B).

### Sociodemographic and clinical factors associated with LTBI screening and TPT acceptance

As presented in Table 3, significant associations were observed between LTBI screening acceptance and several sociodemographic and clinical factors. Department was significantly associated with acceptance, with the highest rate observed in TB diagnosis and treatment department (83.14%), followed by non-TB related departments (75.16%) and administrative departments (71.98%) (*p* = 0.001). Other factors significantly associated with higher acceptance included a history of treating TB patients (*p* < 0.001), close contact with TB patients (*p* = 0.026), a prior history of TB (*p* = 0.031), a prior history of LTBI screening (*p* < 0.001), support for LTBI screening (*p* = 0.019), and support for providing TPT to individuals with LTBI (*p* = 0.027). In contrast, no significant associations were observed for sex, age, educational level, profession, years of working experience, family history of TB, health examination attendance, LTBI knowledge, or care policy (all *p* > 0.05).

**Table 3:** Univariate analysis of factors associated with LTBI screening acceptance.

Characteristics	Variables	Acceptance of LTBI Screening, n (%)		χ^2^	*p*
		Accept (*n* = 804)	Refuse (*n* = 218)		
Sex	Male	186 (23.1)	60 (27.5)	1.807	0.179
	Female	618 (76.9)	158 (72.5)		
Age (years)	≤25	53 (6.6)	8 (3.7)	5.911	0.206
	26–35	380 (47.3)	107 (49.1)		
	36–45	237 (29.5)	69 (31.7)		
	46–55	121 (15.0)	27 (12.4)		
	≥56	13 (1.6)	7 (3.2)		
Department	TB diagnosis and treatment	434 (54.0)	88 (40.4)	13.416	0.001
	Non-TB related departments	239 (29.7)	79 (36.2)		
	Functional office	131 (16.3)	51 (23.4)		
Education level	Master degree or above	70 (8.7)	16 (7.3)	3.537	0.171
	Regular college course	601 (74.8)	176 (80.7)		
	College and below	133 (16.5)	26 (11.9)		
Professional title	Senior	154 (19.2)	31 (14.2)	7.162	0.067
	Intermediate	318 (39.6)	107 (49.1)		
	Junior	297 (36.9)	73 (33.5)		
	other	35 (4.4)	7 (3.2)		
Work experience (years)	≤5	201 (25.0)	43 (19.7)	3.074	0.215
	6–10	195 (24.3)	52 (23.9)		
	≥11	408 (50.7)	123 (56.4)		
Had treated TB patients in the past two years		465 (57.8)	91 (41.7)	19.361	0.000
Had close contact with TB patients without wearing a mask		344 (42.8)	75 (34.4)	4.982	0.026
Family members or relatives diagnosed with TB in 5 years		58 (7.2)	14 (6.4)	0.164	0.685
Had been diagnosed with TB		50 (6.2)	8 (3.7)	2.082	0.149
The units conducted annual health examination		750 (93.3)	207 (95.0)	0.804	0.370
Had a history of TB screening during health examination		611 (76.0)	150 (68.8)	4.659	0.031
Knowledge level of LTBI and TPT	Have no idea at all	28 (3.5)	10 (4.6)	5.881	0.053
	Known a little bit	570 (70.9)	169 (77.5)		
	Familiar with	206 (25.6)	39 (17.9)		
Had a history of LTBI screening		311 (38.7)	22 (10.1)	63.815	0.000
The institution had care policy		336 (41.8)	81 (37.2)	1.525	0.217
Support screening for LTBI in high-risk position		787 (97.9)	207 (95.0)	5.531	0.019
Support TPT for LTBI		665 (82.7)	166 (76.1)	4.864	0.027

Regarding TPT acceptance, acceptance decreased with higher profession rank, from 96.76% in the junior group to 91.53% in the intermediate group and 89.73% in the senior group (*p* = 0.003). Years of working experience also played a significant role: participants with less than 5 years of experience demonstrated higher acceptance (96.76%) compared to those with 6–10 years (94.33%) or more than 11 years (91.34%) (*p* = 0.016). Other factors significantly associated with TPT acceptance included a history of treating TB patients (*p* = 0.041), close contact with TB patients (*p* = 0.026), knowledge level of LTBI (*p* = 0.038), support for LTBI screening in high-risk positions (*p* = 0.005), and support for providing TPT to individuals with LTBI (*p* < 0.001). Conversely, no significant associations were observed with sex, age, department, educational level, family history of TB, health examination attendance, personal history of TB or LTBI screening, or care policy (all *p* > 0.05).

Further analysis revealed that a higher proportion of participants willing to accept TPT had undergone LTBI screening compared to those who refuse TPT (80.0% vs. 72.8%, *p* = 0.027), as shown in Table 4.

**Table 4:** Univariate analysis of factors associated with TPT acceptance.

Characteristics	Variables	Acceptance of TPT, *n* (%)		χ^2^	*p*
		Accept (*n* = 954)	Refuse (*n* = 68)		
Sex	Male	225 (23.6)	21 (30.9)	1.849	0.174
	Female	729 (76.4)	47 (69.1)		
Age (years)	≤25	60 (6.3)	1 (1.5)	6.006	0.199
	26–35	457 (47.9)	30 (44.1)		
	36–45	285 (29.9)	21 (30.9)		
	46–55	135 (14.2)	13 (19.1)		
	≥56	17 (1.8)	3 (4.4)		
Department	TB diagnosis and treatment	479 (50.2)	43 (63.2)	4.812	0.090
	Non-TB related departments	304 (31.9)	14 (20.6)		
	Functional office	171 (17.9)	11 (16.2)		
Education level	Master degree or above	77 (8.1)	9 (13.2)	5.273	0.072
	Regular college course	723 (75.8)	54 (79.4)		
	College and below	154 (16.1)	5 (7.4)		
Professional title	Senior	166 (17.4)	19 (27.9)	14.318	0.003
	Intermediate	389 (40.8)	36 (52.9)		
	Junior	358 (37.5)	12 (17.6)		
	other	41 (4.3)	1 (1.5)		
Work Experience (years)	≤5	236 (24.7)	8 (11.8)	8.313	0.016
	6–10	233 (24.4)	14 (20.6)		
	≥11	485 (50.8)	46 (67.6)		
Had treated TB patients in the past two years		509 (53.4)	47 (69.1)	6.390	0.041
Had close contact with TB patients without wearing a mask		382 (40.0)	37 (54.4)	5.419	0.020
Family members or relatives diagnosed with TB in 5 years		67 (7.0)	5 (7.4)	0.011	0.918
Had been diagnosed with TB		53 (5.6)	5 (7.4)	0.383	0.536
The institution carries out health examination every year		893 (93.6)	64 (94.1)	0.028	0.867
Had a history of TB screening during health examination		714 (74.8)	47 (69.1)	1.094	0.296
Knowledge level of LTBI and TPT	Have no idea at all	36 (3.8)	2 (2.9)	6.545	0.038
	Known a little bit	689 (73.2)	41 (60.3)		
	Familiar with	220 (23.1)	25 (36.8)		
Had a history of LTBI screening		309 (32.4)	24 (35.3)	0.244	0.622
The institution had care policy		394 (41.3)	23 (33.8)	1.469	0.226
Support screening for LTBI in high-risk position		932 (97.7)	62 (91.2)	7.820	0.005
Support TPT for LTBI		802 (84.1)	29 (42.6)	71.663	0.000

### Factors influencing the acceptance of LTBI screening and TPT

Multivariable logistic regression analysis was performed to identify independent predictors of LTBI screening and TPT acceptance, with all variables showing *p* < 0.05 in univariate analyses entered into the regression models.

As shown in Table 5, three factors emerged as independent predictors of LTBI screening acceptance. A prior history of LTBI screening was the strongest predictor (OR = 5.145, 95% CI 3.200–8.275, *p* < 0.05). A history of treating patients with TB was also significantly associated with higher screening acceptance (OR = 1.518, 95% CI 1.054–2.188, *p* < 0.05). Additionally, support for providing TPT to individuals with LTBI independently predicted screening acceptance (OR = 1.497, 95% CI 1.004–2.232, *p* < 0.05). In contrast, department, close contact with TB patients, general TB screening history, and support for LTBI screening were not significantly associated with acceptance after multivariable adjustment (all *p* > 0.05).

**Table 5:** Factors influencing the acceptance of LTBI screening.

Independent variables	B value	*p* value	Odds ratio	95% confidence interval for the odds ratio	
				Lower bound	Upper bound
Department					
TB diagnosis and treatment	0.210	0.387	0.233	0.767	1.984
Non-TB related departments	0.103	0.647	0.109	0.712	1.727
Had treated TB patients in the past two years	0.418	0.025	1.518	1.054	2.188
Had close contact with TB patients without wearing a mask	0.163	0.345	1.177	0.839	1.653
Had a history of TB screening during examination	−0.028	0.873	0.972	0.687	1.375
Had a history of LTBI screening	1.638	0.000	5.145	3.200	8.275
Support screening for LTBI in high-risk position	0.669	0.122	1.952	0.837	4.553
Support TPT for LTBI	0.404	0.048	1.497	1.004	2.232

Regarding TPT acceptance (Table 6), support for providing TPT to individuals with LTBI was the sole independent predictor (OR = 7.110, 95% CI 4.095–12.344, *p* < 0.05). Notably, professional title, years of experience, history of treating TB patients, close contact with TB patients, LTBI knowledge, and support for LTBI screening were not independently associated with TPT acceptance in the multivariable model (all *p* > 0.05).

**Table 6:** Factors influencing the acceptance of TPT.

Independent variables	B value	*p* value	Odds ratio	95% confidence interval for the odds ratio	
				Lower bound	Upper bound
Professional title					
Senior	−0.987	0.377	0.373	0.042	3.3240.521
Intermediate	−0.652	0.549	0.521	0.062	4.406
Junior	0.242	0.823	1.274	0.153	10.639
Work experience (years)					
6–10	0.023	0.965	1.023	0.369	2.842
≥11	−0.020	0.969	0.980	0.364	2.643
Had treated TB patients in the past two years	−0.362	0.224	0.696	0.389	1.248
Had close contact with TB patients without wearing a mask	−0.500	0.071	0.607	0.353	1.044
Knowledge level of LTBI					
Known a little bit	0.448	0.560	1.565	0.347	7.064
Familiar with	−0.010	0.989	0.990	0.212	4.614
Support screening for LTBI in high-risk position	0.339	0.530	1.403	0.488	4.038
Support TPT for LTBI	1.961	0.000	7.110	4.095	12.344

## Discussion

This study provides a comprehensive examination of LTBI screening and TPT acceptance among HCWs in TB-designated institutions in Chongqing, China. Our findings reveal a complex landscape: while willingness to undergo screening (78.7%) and TPT (93.3%) was relatively high, substantial intention–behavior gaps persist, and multivariable analysis identified distinct predictors for LTBI screening versus TPT acceptance. These results carry important implications for intervention design and tuberculosis control policy.

The prior LTBI screening history exerted the strongest effect (OR = 5.145), indicating that HCWs who had personally experienced screening were five times more likely to accept it. This finding aligns with established behavioral models suggesting that past behavior is a reliable predictor of future intentions (22). HCWs who have personally experienced screening may have reduced uncertainty about procedures, lower anxiety regarding potential outcomes, and greater appreciation of the process’s feasibility. From an implementation perspective, ensuring positive initial screening experiences-convenient, minimally disruptive, and accompanied by clear communication may create self-reinforcing cycles of preventive health engagement. This underscores that initial screening encounters are critical junctures; negative experiences could disproportionately deter future participation. History of treating TB patients also independently predicted screening acceptance. According to the Health Belief Model, perceived susceptibility is a core determinant of preventive health behavior; HCWs with direct clinical exposure to infectious patients likely possess more accurate and salient perceptions of their infection risk, motivating screening uptake (23). Notably, department affiliation was not independently associated with acceptance after multivariable adjustment, suggesting that individual-level exposure history may be more proximally related to screening decisions than departmental category per se. This distinction carries practical implications: based on actual exposure patterns rather than departmental categories may yield more efficient screening programs. Support for TPT was also associated with screening acceptance, indicating that favorable attitudes toward preventive treatment extend to screening behaviors. This finding may reflect an underlying “prevention orientation”—HCWs who endorse pharmacological prevention for LTBI are likely those who generally value preventive health services and perceive TB as a condition warranting proactive management. Compared to the prior screening history, although attitude is important, direct personal experience exerts stronger influence on behavioral intentions.

In striking contrast to screening, support for providing TPT to individuals with LTBI emerged as the sole independent predictor of TPT acceptance. This exceptionally strong association suggests that personal acceptance is primarily driven by fundamental beliefs about its value and appropriateness, rather than by demographic characteristics, clinical exposure, or knowledge levels. The magnitude of this effect underscores that attitudes toward TPT, once formed, may be highly consequential for personal health decisions. Notably, professional title and years of working experience were not independently associated with TPT acceptance after multivariable adjustment, despite showing significant univariate associations. This pattern contrasts with findings in general populations (24), and indicates that the inverse relationship between seniority and TPT acceptance may be explained by differential attitudes toward TPT across experience levels. Specifically, senior HCWs in our sample were less likely to support TPT, and this attitudinal difference-rather than seniority per se-appears to drive their lower personal acceptance. Risk Homeostasis Theory offers one explanatory framework, which would predict that experienced HCWs, through extended occupational exposure without developing active disease, may develop attenuated risk perceptions and correspondingly weaker endorsement of preventive interventions (23). From an intervention perspective, these findings suggest that efforts to improve TPT acceptance among senior HCWs should target underlying beliefs about treatment necessity and effectiveness, rather than assuming that knowledge deficits or clinical exposure patterns are primarily responsible for their lower acceptance.

Similarly, knowledge was not independently associated with TPT acceptance in multivariable analysis. This finding, consistent with previous research (25, 26), suggests that while basic awareness of LTBI may facilitate initial consideration of TPT, it is insufficient to drive acceptance in the face of specific concerns. Among the 68 HCWs unwilling to undergo TPT, the predominant concerns were adverse drug reactions (79.4%), perceived long treatment duration (50.0%), and doubted about effectiveness (48.5%). These barriers mirror findings from studies of TPT adherence among close contacts in China, where occurrence of adverse reactions was identified as a significant risk factor for non-adherence (27), and research from Spain demonstrating that shorter regimens were associated with lower non-adherence (28). Collectively, these results reinforce that knowledge-oriented interventions alone are unlikely to substantially improve TPT uptake; addressing specific concerns about treatment safety and burden, as well as fostering positive attitudes toward preventive treatment, may be more effective.

In this study, the high willingness rates observed-78.7% for screening and 93.3% for TPT—must be interpreted alongside the well-documented gap between intention and actual uptake. Longitudinal research among Chinese college students with LTBI found that only 39.1% of those initially willing ultimately received TPT (29), suggesting that stated intentions substantially overestimate subsequent behavior. This pattern is theoretically grounded in the Theory of Planned Behavior, which posits that while attitudes and perceived behavioral control shape intentions, actual implementation is constrained by contextual barriers (22). Our findings support this framework: although attitudes toward TPT strongly predicted personal acceptance, practical obstacles—including time constraints (41.3% among those unwilling to screen), concerns about procedural complexity (57.8%), and cost considerations (22.9% for screening, 13.2% for TPT)—appear to impede translation of intention into action. The divergence between predictors of screening versus TPT acceptance further illuminates this gap. Screening acceptance was shaped by experiential factors (prior screening, clinical exposure). In contrast, TPT acceptance was driven almost exclusively by fundamental attitudes toward TPT, with barriers centered on safety and efficacy concerns. This distinction suggests that different intervention strategies may be required: screening uptake may respond to structural interventions addressing convenience and accessibility, while TPT acceptance requires deeper attitudinal change through targeted communication about treatment safety and effectiveness.

The identification of independent predictors carries specific implications for intervention design. First, the strong effect of prior screening history suggests that ensuring positive initial screening experiences may create virtuous cycles of preventive health engagement. Health systems should invest in making first screening encounters convenient, accompanied by clear communication about procedures and expected outcomes. Second, the role of treating TB patients as an independent predictor indicates that HCWs with direct clinical exposure may be particularly receptive to screening offers; targeting this subgroup for initial implementation efforts could generate early adopters who influence peers through modeling. Third, the primacy of attitudes toward TPT as a predictor of personal acceptance underscores the need for communication strategies that shape fundamental beliefs about preventive treatment—emphasizing its safety, efficacy, and value—rather than merely transmitting factual information.

At the policy level, substantial systemic barriers must be addressed. 59.2% of HCWs reported that their units lacked care policy for TB, and cost concerns were cited by 22.9% of those declining screening and 13.2% declining TPT. Among open-ended recommendations, 19.9% were related to cost issues. The 2024 *Tuberculosis Prevention and Control Action Plan (2025–2035)* issued by Chongqing Municipality signals policy commitment to expanding TPT access, proposing screening of high-risk groups, establishment of TPT clinics, and a target preventive treatment rate of 80% among close contacts by 2030. Building on this framework, we proposes a multi-level implementation strategy encompassing legislative, financial, and institutional interventions. First, mandate LTBI screening through revision of Occupational Health Examination Standards. Second, cover screening costs through basic medical insurance and establish occupational exposure protection funds to subsidize TPT. Third, integrate LTBI interventions into essential public health services and tie hospital infection control budgets to LTBI management performance. Implementation should initially target TB-designated institutions, with gradual expansion based on the monitoring data.

This study represents the first investigation into the knowledge and attitudes toward LTBI screening and TPT among HCWs in TB-designated institutions in China, highlighting critical barriers and opportunities for intervention. However, several limitations warrant consideration. First, the cross-sectional design measured initial willingness but lacked longitudinal follow-up, precluding analysis of decision reversals and temporal attitude shifts. Future studies should employ prospective designs to track the trajectory from intention to actual uptake and identify modifiable factors at each stage, particularly the critical transition from accepting TPT to initiating and completing treatment. Second, by focusing exclusively on TB-designated institutions, our findings may not fully represent HCWs in non-specialized settings where TB awareness and resources are typically lower; replication in diverse healthcare settings is warranted. Third, the sampling approach did not incorporate design effect correction for potential intraclass correlation within institutions. Future surveys should conduct pilot studies to estimate intraclass correlation coefficients prior to main data collection, employ probability-proportional-to-size sampling of institutions, and calculate sample size using Kish’s formula with conservative design effect adjustments.

## Conclusion

In summary, while HCWs in TB-designated institutions report high willingness to undergo LTBI screening and TPT, substantial intention-behavior gaps persist. Prior screening history, clinical exposure to TB patients, and favorable attitudes toward TPT independently predict screening acceptance, while personal TPT acceptance is driven almost exclusively by fundamental beliefs about preventive treatment’s value. These distinct predictors suggest that different intervention strategies are required: screening uptake may respond to structural interventions addressing convenience and accessibility, whereas TPT acceptance requires targeted communication addressing safety and efficacy concerns. Addressing systemic barriers-including inadequate institutional policies, cost obstacles, and procedural complexity-is essential to translate high willingness into sustained uptake. Strengthening health education is insufficient; multifaceted interventions that combine structural support with attitudinal change strategies, targeting high-risk departments and tailoring approaches for experienced HCWs, may enhance compliance with LTBI screening and TPT recommendations.

## Data Availability

The original contributions presented in the study are included in the article/supplementary material, further inquiries can be directed to the corresponding authors.
